# Magnetic and magnetocaloric properties of La_0.55_Bi_0.05_Sr_0.4_CoO_3_ and their implementation in critical behaviour study and spontaneous magnetization estimation

**DOI:** 10.1039/c9ra04141a

**Published:** 2019-08-12

**Authors:** F. Saadaoui, Muaffaq M. Nofal, R. M'nassri, M. Koubaa, N. Chniba-Boudjada, A. Cheikhrouhou

**Affiliations:** Unité de Recherche Matériaux Avancés et Nanotechnologies (URMAN), Institut Supérieur des Sciences Appliquées et de Technologie de Kasserine, Kairouan University BP 471 Kasserine 1200 Tunisia saadaoui.fadhel80@gmail.com rafik_mnassri@yahoo.fr; Department of Mathematics and General Sciences, Prince Sultan University P. O. Box 66833 Riyadh 11586 Saudi Arabia; LT2S Lab (LR16 CNRS 01), Digital Research Center of Sfax Sfax Technopark, Cité El Ons, B.P. 275 3021 Tunisia; Institut NEEL B. P.166 38042 Grenoble Cedex 09 France

## Abstract

In this work, we present the results of the magnetic, critical, and magnetocaloric properties of the rhombohedral-structured La_0.55_Bi_0.05_Sr_0.4_CoO_3_ cobaltite. Based on the modified Arrott plot, Kouvel–Fisher, and critical isotherm analyses, we obtained the values of critical exponents (*β*, *γ*, and *δ*) as well as Curie temperature (*T*_C_) for the investigated compound. These components were consistent with their corresponding values and they were validated by the Widom scaling law and scaling theory. The obtained critical exponents were close to the theoretical prediction of the mean-field model values, revealing the characteristic of long-range ferromagnetic interactions. The magnetic entropy, heat capacity, and local exponent *n*(*T*, *μ*_0_*H*) of the La_0.55_Bi_0.05_Sr_0.4_CoO_3_ compound collapsed to a single universal curve, confirming its universal behaviour. The estimated spontaneous magnetization value extracted through the analysis of the magnetic entropy change was consistent with that deduced through the classical extrapolation of the Arrott curves. Thus, the magnetic entropy change is a valid and useful approach to estimate the spontaneous magnetization of La_0.55_Bi_0.05_Sr_0.4_CoO_3_.

## Abstract 2

In this work, we present the results of the magnetic, critical, and magnetocaloric properties of the rhombohedral-structured La_0.55_Bi_0.05_Sr_0.4_CoO_3_ cobaltite.

## Graphical abstract

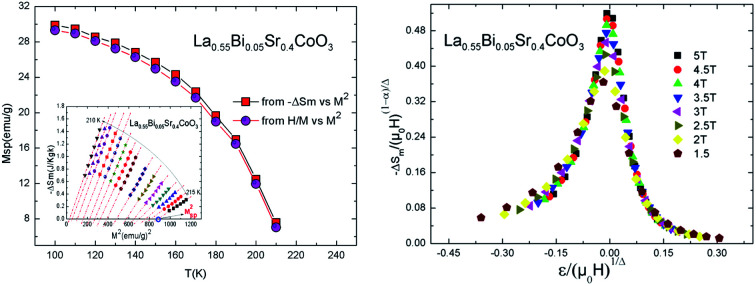

## 1. Introduction

As a member of perovskite oxides, cobaltites are important agile and multifunctional materials that are very promising for several applications, including high-temperature oxygen separation membranes, cathodes in solid oxide fuel cells (SOFCs), magnetic storage, and magnetic refrigeration.^1–3^ The Co-based sister compounds of manganites have been less intensively studied.^4–6^ It is well-known that the similar Hund's rule exchange energy and crystal-field energy both lead to an additional spin-state degree of freedom in cobaltites, which results in close competition between the multiple ground states. This, in turn, leads to phenomena such as magnetoelectronic phase separation, colossal magnetoresistance, and magnetocaloric effect. The spin state (ST) of cobalt ions is very sensitive to the application of external stimuli such as magnetic and electrical fields, temperature, pressure, or compositional doping.^7,8^ This sensitivity is due to the very small energy difference between the t_2g_ and e_g_ levels. Consequently, the state of Co can be presented in a low-spin (LS), intermediate-spin (IS), or high-spin (HS) state.^4,5,8^ This ability of Co to exist in several STs given a principal property distinguishes the cobalt oxides from other transition metals such as manganese and makes the physical phenomena observed in the cobalt oxides very complex, due to which they have not been entirely comprehended so far. The complexity of STs provides promising opportunities for basic science as well as electronic applications, inducing a multifunctional characteristic to cobalt oxides. The latter property derives from the fact that the crystal-field splitting of the 3d energy level of Co ions in cobalt oxides is of the same order of magnitude as the Hund's rule intra-atomic exchange energy and 3d orbital bandwidth.^9^

Physical effects such as magnetoresistance and magnetocaloric effects observed in manganites^10,11^ and cobaltites^3,12–14^ have been the subject of several investigations in the last few years. Double exchange (DE), phase separation (PS), Jahn–Teller distortion (JT), and Griffiths phase (GP) have been found to explain the aforementioned effects. Moreover, these compounds are also interesting for applications since they present low costs and longer usage times. This family of materials can be easily elaborated, and grain growth can be achieved to the desired size; moreover, they possess tunable Curie temperature and high chemical stability. La-based cobaltite is one of the perovskite oxides and it shows a wide variety of physical properties with relatively high *T*_C_ values. Like manganese, iron, and copper, cobalt exhibits various possible oxidation states (Co^2+^, Co^3+^, and Co^4+^) and several types of coordinations (tetrahedral, pyramidal, and octahedral). Consequently, cobalt oxides offer a wide gamut of opportunities for the creation of many frameworks involving a mixed valence state of cobalt. Similar to manganites, the substitution of La^3+^ by Sr^2+^ in La_1−*x*_Sr_*x*_CoO_3_ converts an adapted number of Co^3+^ to Co^4+^, introducing a predominantly ferromagnetic (FM) order due to the DE interactions between Co^3+^ and Co^4+^ ions.^15^ In addition, the substitution at the A-site of cobaltite oxides induces changes in the chemical internal pressure that locally affects the Co–O–Co networks and easily modifies the ST of cobalt ions due to the fact that crystal-field splitting is very sensitive to changes in the Co–O–Co angle and Co–O distance. Therefore, Co^3+^ adopts three possible STs, namely, LS, IS, and HS, whereas Co^4+^ usually exhibits only the LS.

It is believed that doped cobaltites are not a homogeneous ferromagnet. This inhomogeneity might affect the cooperative behavior of the Co sublattice, and therefore, the nature of FM–paramagnetic (PM) phase transition and the class of universality of this magnet.^16^ Therefore, the analysis of critical exponents (*β*, *γ*, and *δ*) of a magnetic system can yield valuable information about the magnetic phase transitions and can be classified into different universal classes, such as mean field, 3D Ising, and 3D Heisenberg, depending on the exponent values. Recently, the theory of critical phenomena justified the existence of a universal MCE behavior in materials exhibiting second-order magnetic phase transitions.^17^ Recent studies have revealed the impact of Bi^3+^ substitution on several properties in La-based manganites.^18–20^ It is believed that the BiMnO_3_ system is a special and promising compound, which exhibits multiferroic properties where phases like ferroelectric, FM, and ferroelastic coexist in this oxide. Earlier reports have suggested that Bi doping in manganite systems exhibit a high charge ordering temperature^21^ and that Bi doping in LaCaMnO_3_ exhibits excellent MCE properties with high efficiency.^18,20^ However, there are relatively few investigations that deal with the investigation of critical and MCE behaviors in Bi-doped La-cobaltite system. In order to understand both these behaviors in rare-Earth cobaltites keeping in mind the abovementioned factors, we have performed a comprehensive investigation of the structural, magnetic, critical, and MCE properties of a small amount (5%) of Bi-doped La_0.55_Bi_0.05_Sr_0.4_CoO_3_ samples. For a better understanding of the nature of magnetic transition and MCE properties of La_0.55_Bi_0.05_Sr_0.4_CoO_3_, we obtained the universal curves of magnetic entropy changes. Further, we have made an effort to estimate the spontaneous magnetization (*M*_SP_) from magnetic entropy changes and then compared the results with the standard extrapolation of Arrott plots. The present investigation is an attempt to fill this gap to a certain extent and to comprehensively explore the magnetic transition nature of a La_0.55_Bi_0.05_Sr_0.4_CoO_3_ system.

## 2. Experimental details

The ceramic with a nominal composition of La_0.55_Bi_0.05_Sr_0.4_CoO_3_ was synthesized by the solid-state reaction method. The precursors of La_2_O_3_ (Aldrich 99.9%; USA), Bi_2_O_3_ (Aldrich 99.9%; USA), SrCO_3_ (Aldrich 99.9%; USA), and Co_3_O_4_ (Aldrich 99.9%; USA) were mixed in an agate mortar with the desired proportions according to the La_0.55_Bi_0.05_Sr_0.4_CoO_3_ system. Then, the obtained powder was heated at 800 °C for 24 h. After cooling to the ambient temperature, the bulk was ground and pressed into pellets and then sintered at 900, 1000, 1100, and 1200 °C for 24 h with intermediate regrinding and repelling to ensure homogenization. Finally, the obtained disk-shaped samples were slowly cooled to room temperature in air. As the sample was exposed to air, it consequently became stoichiometric with respect to oxygen.^22,23^ The X-ray diffraction (XRD) pattern was recorded at room temperature on a PANalytical X'PERT Pro MPD diffractometer using *θ*/2*θ* Bragg–Brentano geometry with diffracted beam monochromatized CuKα radiation (*λ* = 1.5406 Å). The diffraction patterns were collected at steps of 0.017° over the angle range of 20–80°. Rietveld refinement was performed to determine the structural parameters by using the FullProf software. A vibrating-sample magnetometer developed at NEEL Institute was used to investigate the thermomagnetic properties of the sample. The temperature and field dependencies of the magnetization, *M*(*T*, *μ*_0_*H*), were recorded in a temperature range around the *T*_C_. To accurately extract the critical exponents of the sample, the corrected magnetic isotherms were measured in the range of 0–5 T and within a temperature interval of 2 K in the vicinity of *T*_C_.

## 3. Scaling analysis

For continuous phase transition, near *T*_C_, the critical behavior for a second-order magnetic phase transition can be investigated *via* a series of critical exponents (*β*, *γ*, and *δ*). According to the scaling hypothesis, these exponents are expressed as follows:^24,25^1
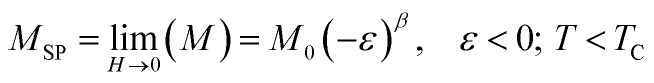
2
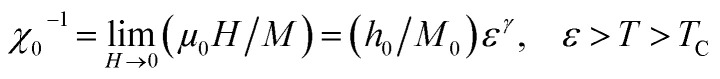

3 *M* = *D*(*μ*_0_*H*)^1/*δ*^, *ε* = 0; *T* = *T*_C_

where *ε* = (*T* − *T*_C_)/*T*_C_ is the reduced temperature. *M*_0_, *h*_0_, and *D* are the critical amplitudes; *β* (associated with *M*_SP_), *γ* (associated with magnetic susceptibility *χ*_0_^−1^), and *δ* (associated with the field-dependent magnetization at *T*_C_) are the critical parameters.

The critical exponents (*β*, *γ*, and *δ*) should obey the scaling equations.^24,25^ A formulation was used in this work, which was based on the scaling equations of state. In the asymptotic critical region and according to the scaling equations, the magnetic equation can be expressed as follows:

4 *M*(*μ*_0_*H*, *ε*) = *ε*^*β*^*f*_±_(*μ*_0_*H*/*ε*^*β*+*γ*^)

where *f*_±_ denote regular analytic functions with *f*_−_ denoting the FM state for *T* below *T*_C_ and *f*_+_ denoting the PM state for *T* above *T*_C_. Eqn (4) shows that for true scaling relations and for the right choice of *β*, *γ*, and *δ*, *M*(*μ*_0_*H*, *ε*)*ε*^−*β*^*vs. μ*_0_*Hε*^−(*β*+*γ*)^ yields two universal curves of temperature *T* above and below *T*_C_.

## 4. Results and discussions

**Fig. 1:**
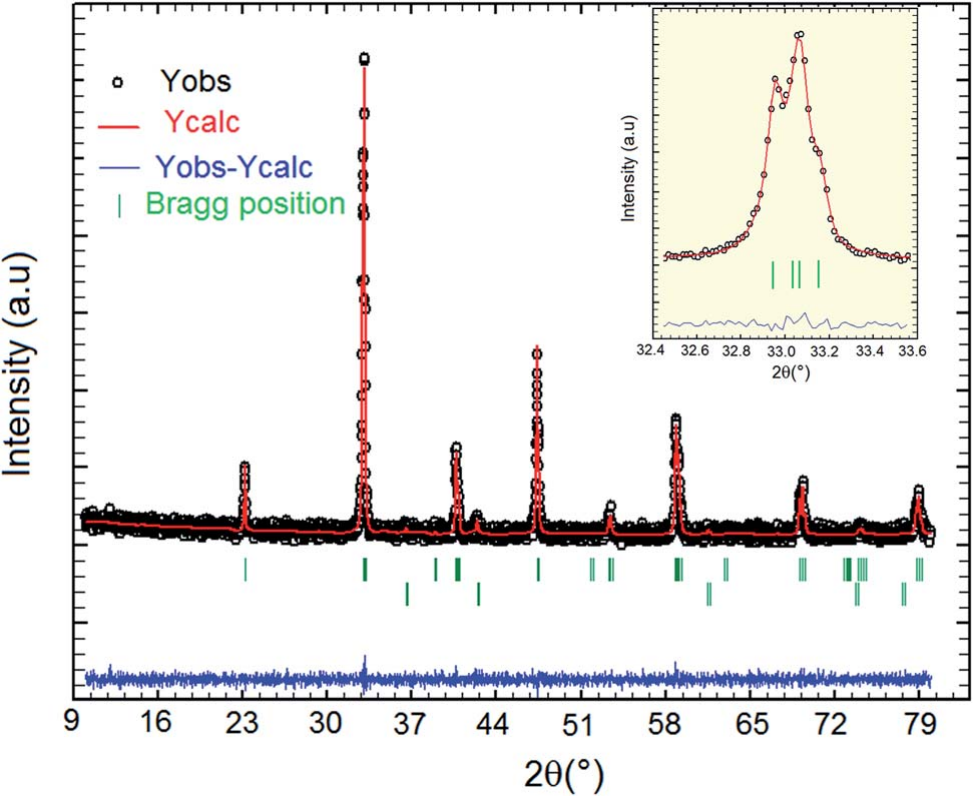
Room-temperature XRD pattern for the La_0.55_Bi_0.05_Sr_0.4_CoO_3_ sample.

The XRD *θ*–2*θ* patterns measured at room temperature show that La_0.55_Bi_0.05_Sr_0.4_CoO_3_ can be refined in the perovskite structure with a rhombohedral structure with the *R*3̄*c* space group. This demonstrates that the substitution of La by 5% Bi does not induce the relevant effect on the crystal structure as compared to those in the undoped La_0.5_Sr_0.4_CoO_3_ compound prepared by the solid-state reaction method^26^ or sol–gel technique.^14^ The structural information of the prepared sample is obtained after fitting the XRD data using the Rietveld refinement technique by means of the FullProf software.^27,28^ During the initial stages of refinement, only the following parameters were changed: scale factor, background coefficients, unit-cell parameters, full-width parameters, sample displacement, and peak asymmetry. First, only the scale factor was refined; then, the remaining parameters were gradually included in the successive least-squares cycles. The background was fit using a third-order polynomial, and the observed peak shapes were approximated by using a pseudo-Voigt profile function, limited to ten full-widths on each side of the peak maximum. Fig. 1 shows the XRD diffractogram for the investigated sample, where the open circle dots represent the measured XRD reflections and the solid lines denote the Rietveld-refined results. Evidently, a marginal difference between the measured spectra and refined ones can be observed. The quality of refinement is evaluated through the goodness of the fit indicator *χ*^2^, which is equal to 1.98. From Fig. 1, it is clear that a small secondary phase can be observed, which can be attributed to the presence of the CoO impurity. This impurity is identified with the X'Pert HighScore Plus software. It is interesting to note that the most intense peak in the XRD spectra for La_0.55_Bi_0.05_Sr_0.4_CoO_3_ exhibits a double peak, representing the rhombohedral phase. The intense peak is shown in the inset of Fig. 1. The lattice parameters are found to be *a* = *b* = 5.415(7), *c* = 13.326(9) Å, and unit cell volume *V* = 338.51 Å^3^ for La_0.55_Bi_0.05_Sr_0.4_CoO_3_. For the undoped compound prepared by using the conventional solid-state reaction method, the lattice parameters can be determined as *a* = *b* = 5.436(3), *c* = 13.226(3) Å, and unit cell volume *V* = 338.49(1) Å^3^.^26^ It is clear that the lattice parameter “*c*” and unit cell volume marginally increase with the addition of 5% Bi content, while parameter “*a*” slightly decreases with the addition of 5% Bi content. This almost-perfect match can be explained considering the similar ionic radii of La^3+^ ions (1.16 Å, 8-coordinate) and Bi^3+^ ions (1.17 Å, 8-coordinate).^29^

**Fig. 2:**
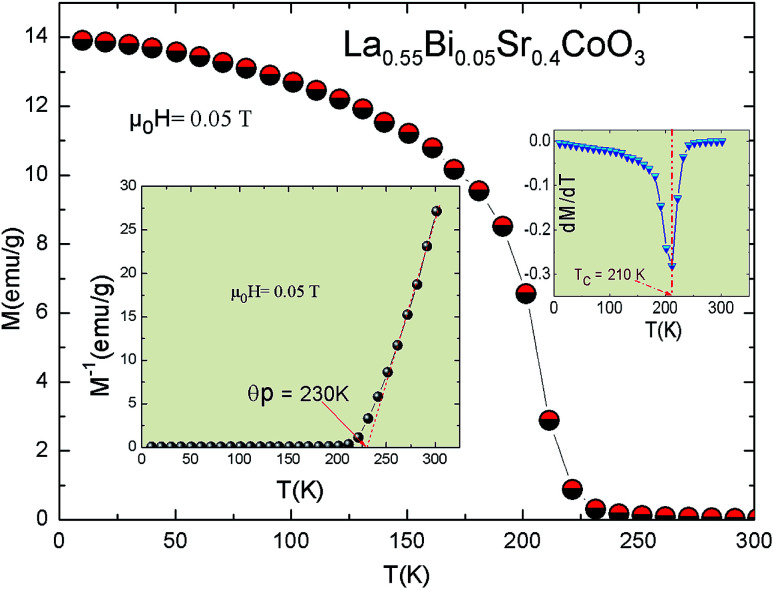
Magnetization measurements as a function of temperature for the La_0.55_Bi_0.05_Sr_0.4_CoO_3_ sample under 0.05 T. Insets: d*M*/d*T* as a function of temperature and temperature dependence of the inverse of magnetic susceptibility. The red line presents a linear fit at higher temperatures.

In order to understand the general magnetic behavior and to estimate *T*_C_, low-field magnetization *vs.* temperature (*M*(*T*)) is obtained in the field-cooled (FC) mode for the La_0.55_Bi_0.05_Sr_0.4_CoO_3_ sample. Fig. 2 shows the *M*(*T*) curve under an applied magnetic field of 0.05 T. This curve exhibits a sharp FM–PM phase transition, where *T*_C_, defined from the inflexion point of the d*M*/d*T vs. T* curve (inset, Fig. 2), is found to be 210 K. Here, *T*_C_ is suppressed by 20 K as compared to undoped La_0.5_Sr_0.4_CoO_3_.^14^ It is noteworthy that even a higher value of *T*_C_ = 237 K was found by T. A. Ho *et al.*^26^ for a sample prepared under different conditions. This *T*_C_ suppression correlates with the competition between the DE interaction and superexchange interactions altered by the incorporation of Bi ion in the Co–O–Co networks. The observed *M*(*T*) curve reveals a strong variation in the magnetization around *T*_C_, which indicates that there is possibly a large magnetic entropy change around the magnetic transition.^11,30^ When showing the inverse of the magnetic susceptibility (1/*χ*–1/*M*) curve in the PM state (insets, Fig. 2), a linear behavior with temperature is observed above *T*_C_, which can be fitted with the Curie–Weiss law:

5 *χ* = *C*/(*T* − *θ*_P_)

where *θ*_P_ is the PM *T*_C_ and *C* is the Curie constant. The obtained *θ*_P_ is determined to be 230 K, which is higher than *T*_C_, where Δ*T* = *θ*_P_ − *T*_C_ = 20 K. This *θ*_P_ value is smaller than the one observed in the undoped compound (245 K).^26^ The decrease in *θ*_P_ with 5% Bi doping can be explained by considering the DE mechanism in cobaltites. It is known that Bi^3+^, with the 6s^2^ lone pair, is a highly polarizable ion and it induces local distortions in the Co–O–Co bond angles, resulting in a decrease in the DE interaction strength. For our sample, the positive values of *θ*_P_ and Δ*T* reveal the existence of a FM exchange interaction between the nearest neighbors in the PM region and confirm the presence of a magnetic inhomogeneity.^31,32^ Similar results are observed in several manganites^33,34^ and cobaltite perovskites, such as La_0.6_Sr_0.4_CoO_3_.^14,26^

**Fig. 3:**
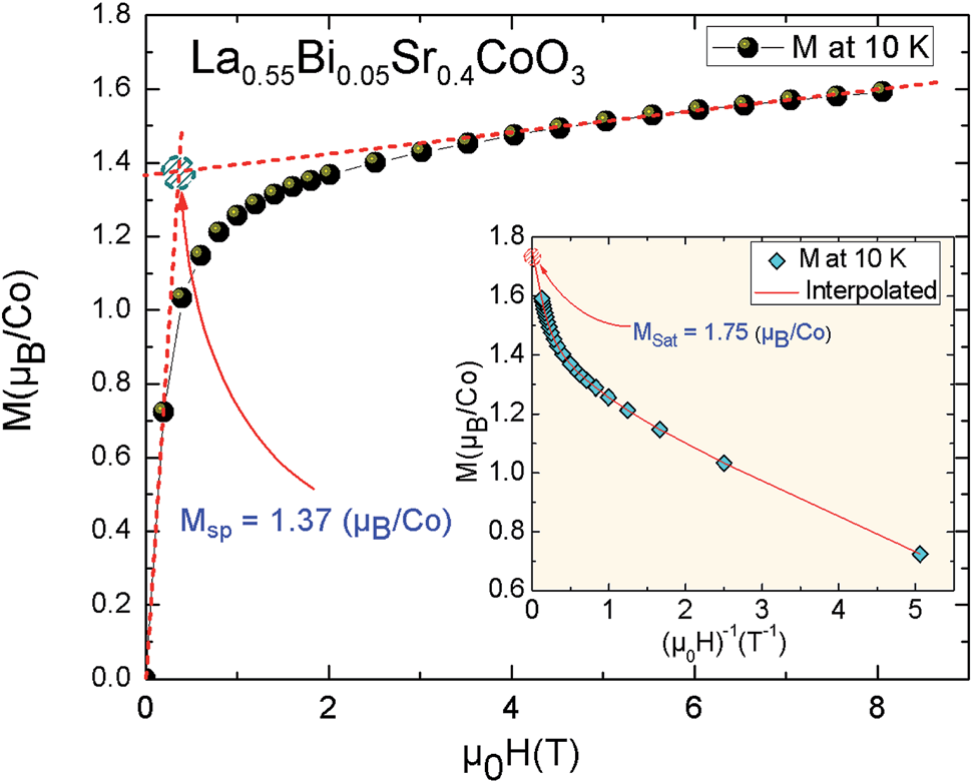
Variation in magnetization as a function of the applied magnetic field for the La_0.55_Bi_0.05_Sr_0.4_CoO_3_ sample at 10 K. Inset: determination of the saturation magnetization of the sample at 10 K.

Fig. 3 shows the magnetization *vs.* applied magnetic field curve up to 8 T, *M*(*μ*_0_*H*), recorded at 10 K. This curve shows the typical FM nature of La_0.55_Bi_0.05_Sr_0.4_CoO_3_. The magnetization sharply increases with the applied magnetic field for *μ*_0_*H* = 1 T, but does not saturate even up to a field of 8 T. Such results confirm the existence of FM clusters, which, in cobaltites, are related to the presence of a spin disorder in the system, with indifferent STs of Co^3+^ and Co^4+^ ions.^15^ Further, the *M*(*μ*_0_*H*, *T* = 10 K) curve can be used for the evaluation of the characteristic magnetization values by means of the procedures described elsewhere.^35^ Namely, the saturation magnetization (*M*_sat_) calculated from the *M vs.* 1/*H* plot at 1/*H* → 0 (inset, Fig. 3). The obtained *M*_sat_ values reach 1.75 *μ*_B_/Co in the same order as the other perovskite systems.^33,36,37^ At low temperatures, the spontaneous magnetization *M*_sp_(exp) determined by the extrapolation of *M*(*μ*_0_*H*) from the high field to zero field is determined to be 1.37 *μ*_B_/Co (inset, Fig. 3).

**Fig. 4:**
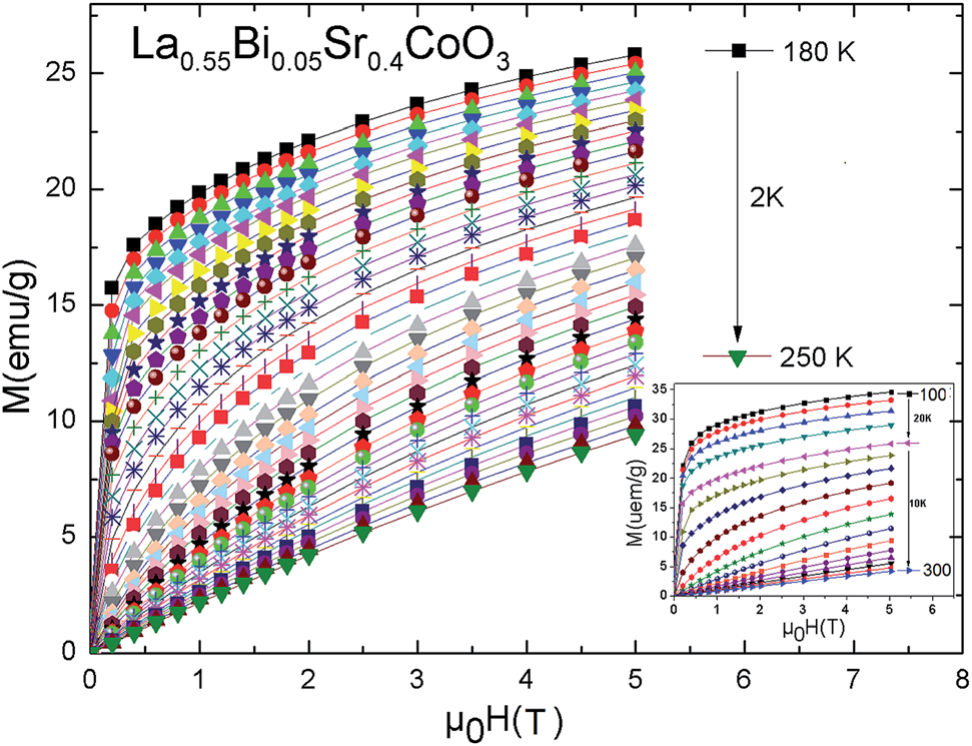
Isothermal magnetization curves at various temperatures for the La_0.55_Bi_0.05_Sr_0.4_CoO_3_ sample.

Fig. 4 shows the isothermal curves *M*(*μ*_0_*H*) over a wide range of temperatures and external fields up to 5 T. These curves are used to determine the changes in the magnetic entropy (−Δ*S*_m_) and critical exponents (*β*, *γ*, and *δ*). From the data, it is clear that the La_0.55_Bi_0.05_Sr_0.4_CoO_3_ sample shows FM behavior below *T*_C_ and PM behavior considerably above *T*_C_. The *M*(*μ*_0_*H*) curves of the sample in the temperature range of 100–300 K are shown in the inset of Fig. 4. At lower temperatures, the *M*(*μ*_0_*H*) curve reveals that the magnetizations rise sharply in weak applied magnetic fields and then progressively increase with the *μ*_0_*H* value. However, a diminution of magnetization with increasing temperature is clearly observed. Further, it is clear that at *T*_C_, the investigated material transits from the FM state to the PM state. This transition is due to the magnetic disorder established as the temperature increases. In this case, the deflection of magnetic momentum occurs, and hence, the total magnetic moment of the entire system decreases and compound magnetization gets diminished. Therefore, once the temperature reaches *T*_C_, the thermal motion of the molecules of the material affects the ordered spin at the zero field and the PM behavior is observed instead of the FM behavior.

**Fig. 5:**
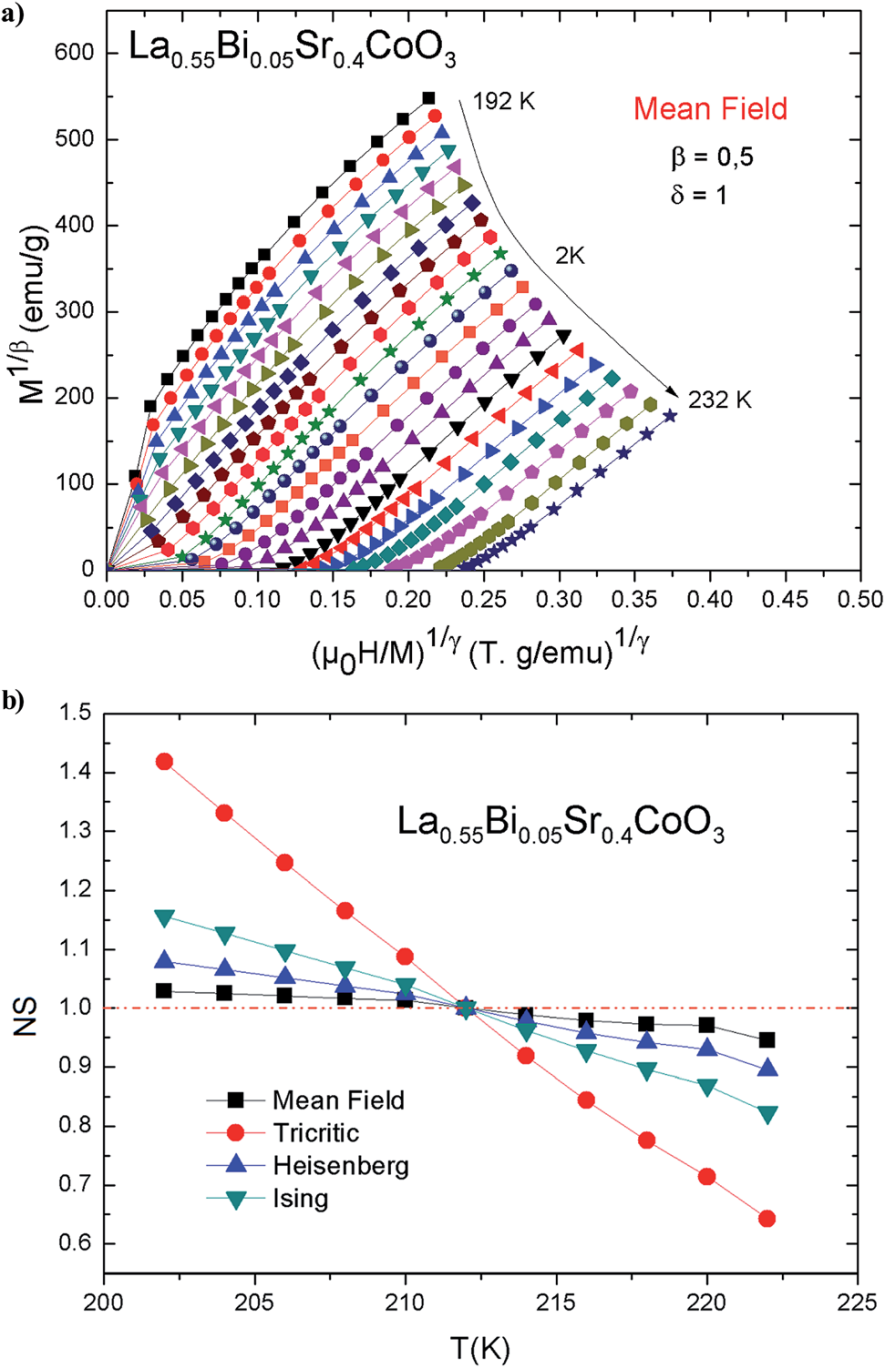
(a) *M*^2^*vs. μ*_0_*H*/*M* isotherms for La_0.55_Bi_0.05_Sr_0.4_CoO_3_; (b) NS as a function of temperature for La_0.55_Bi_0.05_Sr_0.4_CoO_3_.

In order to determine the type of magnetic phase transition in the vicinity of *T*_C_, Fig. 5a shows the Arrott curves (*M*^2^–*μ*_0_*H*/*M*) for the prepared La_0.55_Bi_0.05_Sr_0.4_CoO_3_ compound. In these curves, it is assumed that the critical exponents follow the mean-field theory (MFT), where *β* = 0.5 and *γ* = 1.^38^Fig. 5a shows that in the low-field region, the nonlinear and curvature characters in *M*^2^–*μ*_0_*H*/*M* parts at *T* > *T*_C_ and *T* < *T*_C_ are driven toward two opposite directions. The latter phenomenon is essentially due to the misaligned magnetic domains, which reveal the FM–PM separation and indicate that the values of *β* = 0.5 and *γ* = 1 are inaccurate.

**Fig. 6:**
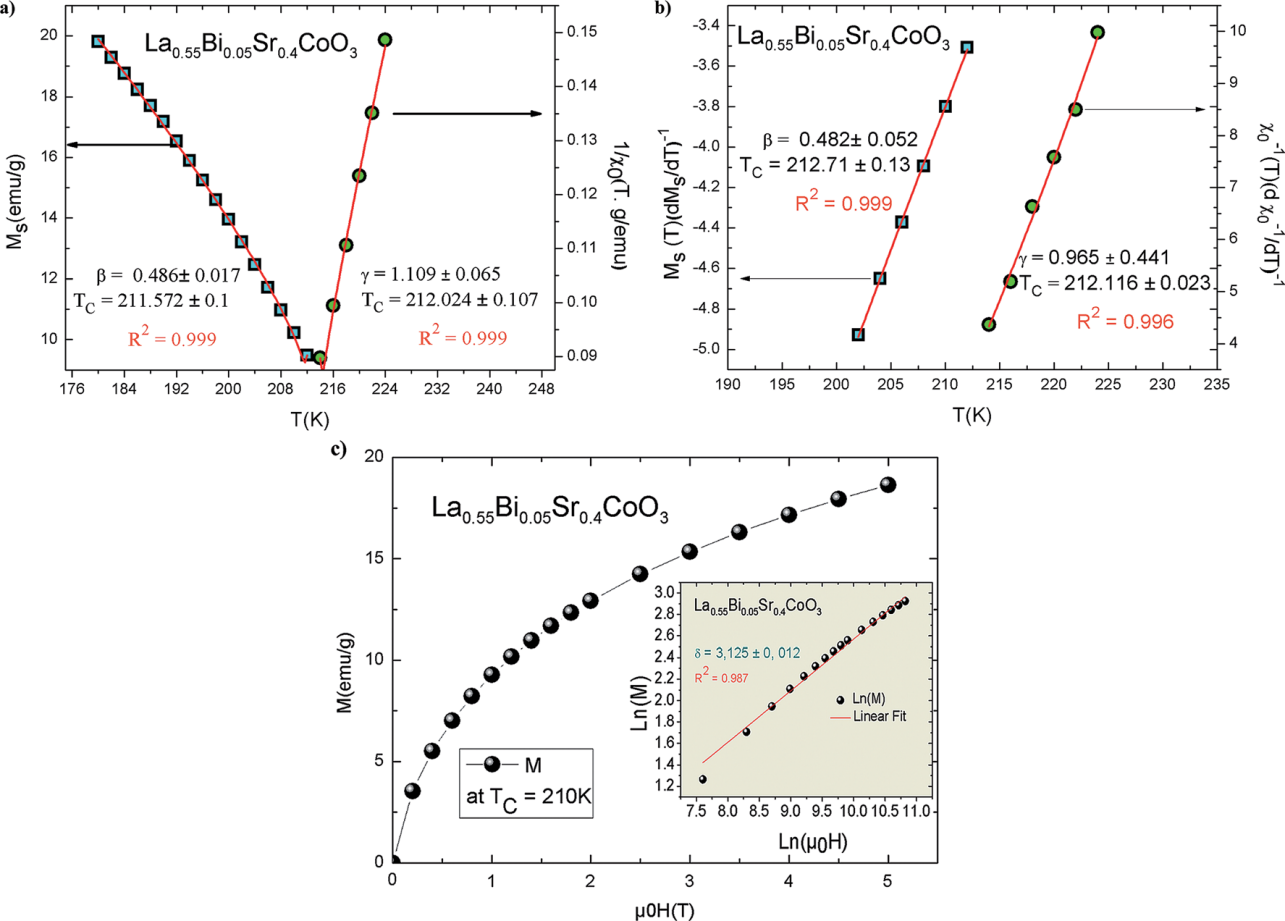
(a) Spontaneous magnetization (left axis) and inverse initial susceptibility (right axis) *vs.* temperature for La_0.55_Bi_0.05_Sr_0.4_CoO_3_. (b) *K*–*F* construction for determining the critical exponents and *T*_C_; solid lines are the fitted data. (c) Critical isotherm of *M vs. μ*_0_*H* for La_0.55_Bi_0.05_Sr_0.4_CoO_3_. Inset: Ln–Ln scale for *M vs. μ*_0_*H*.

The characteristics of the magnetic phase transition in the La_0.55_Bi_0.05_Sr_0.4_CoO_3_ cobaltite can be determined by assessing the feature of the Arrott plots around *T*_C_. In our case, no inflection or negative slope is observed as a signature of the metamagnetic transition above *T*_C_, indicating the nature of the second-order phase transition (SOPT). This is in agreement with those observed in earlier studies.^14,39,40^ In general, for SOPT, its thermodynamic function can be expressed in the form of a power law with the aforementioned critical exponents, namely, *β*, *γ*, and *δ*. This transition obeys the following asymptotic relations. By using eqn (1) and (2), as well as the so-called Arrott–Noakes equation of state 
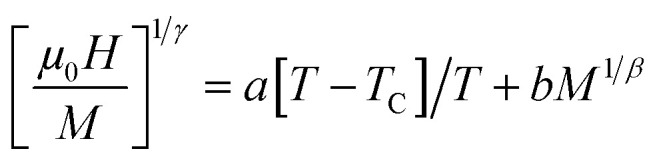
 (where *a* and *b* are constant parameters), we can obtain the values of *β* and *γ*; this approach is known as the modified Arrott plots (MAPs).^41^ Therefore, to determine the correct values of *β* and *γ*, MAPs should be used. In this context, it is possible to initially use the tri-critical mean-field model (*β* = 0.25 and *γ* = 1), 3D Ising model (*β* = 0.325 and *γ* = 1.24), 3D Heisenberg model (*β* = 0.365 and *γ* = 1.386), and mean-field model (*β* = 0.5 and *γ* = 1) to construct tentative Arrott plots and then the select the best one to be the initial Arrott plot for fitting the data. The so-called normalized slope (NS) defined as NS = *S*(*T*)/*S*(*T*_C_) at the critical point can be used for effecting further comparisons. Since the modified Arrott plots are a series of parallel lines, the NS of the most satisfactory model should be close to 1 (unity) regardless of temperature.^42^ As shown in Fig. 5b, the mean-field model provides an NS value closest to 1 in the temperature range under investigation. The latter model is the best one to determine the critical exponents as well as to describe the material. The temperature dependencies of *χ*_0_^−1^(*T*) and *M*_SP_(*T*) are shown in Fig. 6a; eqn (1) and (2) are used for fitting these data. These fits yielded the critical parameters as *β* = 0.486 ± 0.017 with *T*_C_ = 211.572 ± 0.1 K and *γ* = 1.109 ± 0.065 with *T*_C_ = 212.024 ± 0.107 K. These results are very close to the exponents of the mean-field model (*β* = 0.5 and *γ* = 1). It is evident that the obtained value of *T*_C_ agrees well with that obtained from the *M*(*T*) curve.

Alternatively, in order to more accurately determine the *β*, *γ*, and *T*_C_ parameters, we can use the Kouvel–Fisher (KF) method^43,44^ expressed as6
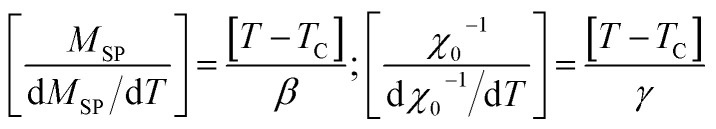


According to the above equations, the 1/*β* and 1/*γ* slopes are obtained by linear fitting and the value of *T*_C_ is obtained from the intercepts on the temperature axis. The results of the best fits are shown in Fig. 6b, where (*β* = 0.482 ± 0.052, *T*_C_ = 212.71 ± 0.13 K) and (*γ* = 0.965 ± 0.441, *T*_C_ = 212.116 ± 0.023 K) for the La_0.55_Bi_0.05_Sr_0.4_CoO_3_ compound. It is worth noting that these new values are also in agreement with those obtained by the MAPs, which indicates that the estimated values are self-consistent and unambiguous.

The third critical exponent, namely, *δ*, is determined from the Widom scaling relation, *i.e.*, *δ* = 1 + *γ*/*β*. Here, *δ* is calculated to be 3.281 ± 0.084 as obtained by the MAPs and 3.002 ± 0.493 as obtained by KF. Fig. 6c shows the magnetic isotherm *M*(*μ*_0_*H*; *T*_C_ = 210 K) and the same plot on the Ln–Ln scale. The Ln(*M*) *vs.* Ln(*μ*_0_*H*) curve would be a straight line with a slope of 1/*δ*. The values of *δ* obtained through the MAP and KF methods are close to that obtained from the fitting of the isotherm at *T* = *T*_C_ to eqn (3) (*δ* = 3.125 ± 0.012). Moreover, we note that these values of the critical exponents are in good agreement with those obtained from the mean-field model (*β* = 0.5, *γ* = 1, and *δ* = 3). When compared with La_0.5_Sr_0.4_CoO_3_, it is evident that the critical exponents of the La_0.55_Bi_0.05_Sr_0.4_CoO_3_ sample are different from those observed for the undoped sample. It has been reported that the critical exponents for La_0.5_Sr_0.4_CoO_3_ prepared by the sol–gel process are in good agreement with those predicted by the 3D Heisenberg model.^14^ However, many inconsistent results have been reported on La_0.6_Sr_0.4_CoO_3_ made by the solid–solid reaction. For example, T. A. Ho *et al.* found a complex scenario in the La_0.5_Sr_0.4_CoO_3_ sample.^26^ The *β* value of their sample was located between those expected from the mean-field model and 3D Heisenberg model, while the *γ* value is close to the value obtained from the 3D Ising model. Consequently, it is clear that the replacement of lanthanum by 5% Bi in the La_0.6_Sr_0.4_CoO_3_ sample induced a long-range magnetic order and modified the class of universality of the sample. In general, these characteristics are related to the differences in the sintering temperatures, preparation routes, particle sizes and shapes, and local geometric structures, resulting in various inhomogeneities and magnetocrystalline anisotropies. In our case, the obvious differences in the class of universalities of La_0.55_Bi_0.05_Sr_0.4_CoO_3_ and the parent compound are due to the influence of the replacement of La^3+^ ion (with zero magnetic moment) by Bi^3+^ ion (with nonzero magnetic moment). Therefore, the insertion of 5% Bi may contribute toward the magnetic interactions along with Co^3+^ and Co^4+^ ions and causes a difference in the critical behavior in the aforementioned samples.

In addition, the scaling equation stipulates that the *M*(*μ*_0_*H*, *T*) data in the critical region obeys the scaling relation expressed as^45^

**Fig. 7:**
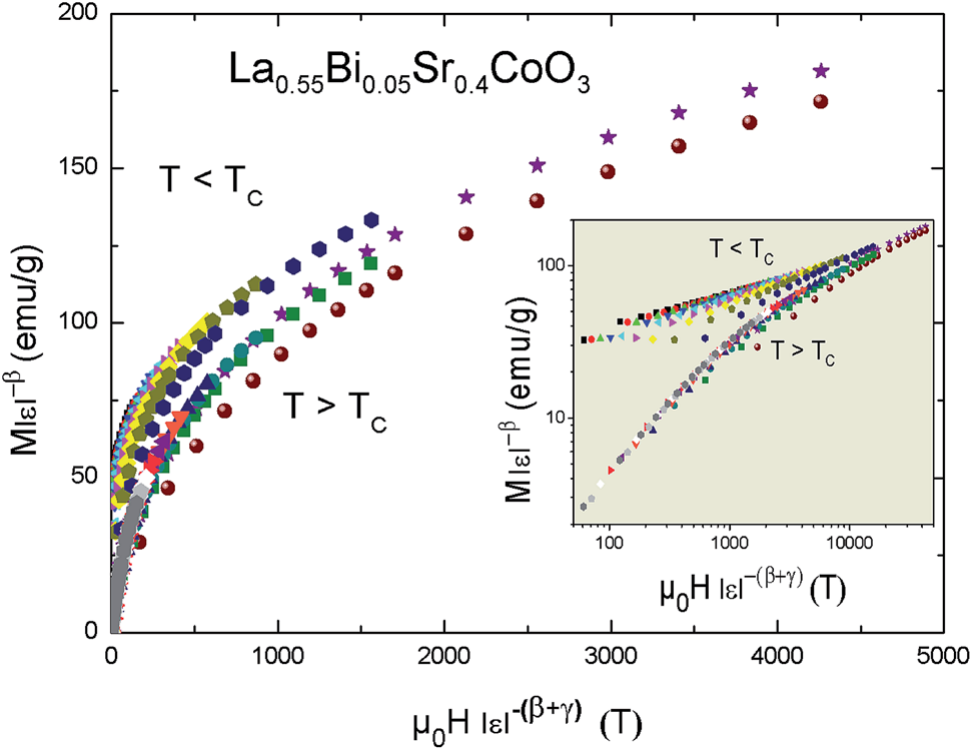
Scaling plot of *M*|*ε*|^−*β*^*vs. μ*_0_*H*|*ε*|^−(*β*+*γ*)^ for the La_0.55_Bi_0.05_Sr_0.4_CoO_3_ compound at temperature *T* < *T*_C_ and *T* > *T*_C_.

7 *M*(*μ*_0_*H*, *ε*) = *ε*^*β*^*f*_±_(*μ*_0_*H*/*ε*^*β*+*γ*^)

where *f*_+_ and *f*_−_ are the analytical functions for *T* > *T*_C_ and *T* < *T*_C_, respectively. Eqn (8) shows that *M* (*μ*_0_*H*, *ε*)*ε*^−*β*^*vs. μ*_0_*Hε*^−(*β*+*γ*)^ yields two distinct curves: one for *T* > *T*_C_ and the other for *T* < *T*_C_. By using the correct (KF)-*β* and (KF)-*γ* values for La_0.55_Bi_0.05_Sr_0.4_CoO_3_, the *M*/|*ε*|^*β*^*vs. H*/|*ε*|^(*β*+*γ*)^ curves are plotted, as shown in Fig. 7. Evidently, they collapse onto two sides: one above *T*_C_ and the other below *T*_C_. The obedience of the scaling equation over the entire range of normalized variables proves that the critical exponents and *T*_C_ are reasonably accurate and unambiguous with the scaling theory.

The isothermal magnetic entropy change (Δ*S*_m_), which results from the spin ordering under the influence of a magnetic field, can be obtained from the *M*(*μ*_0_*H*) curves at various temperatures according to the classical thermodynamic theory based on Maxwell's relations by using the following expression:^46^8
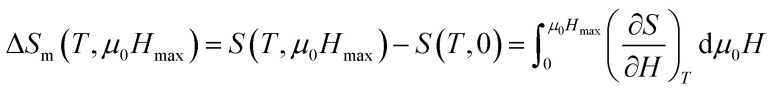


In this work, the magnetization measurements are made under discrete magnetic fields and temperature intervals. Therefore, the magnetic entropy change can be approximately given by9
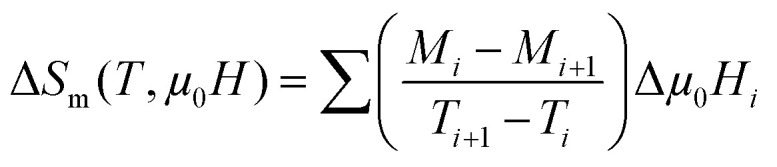


In this equation, *M*_*i*_ and *M*_*i*+1_ are the experimental values of magnetization measured at temperatures *T*_*i*_ and *T*_*i*+1_, respectively, under the applied magnetic field *μ*_0_*H*_*i*_.

**Fig. 8:**
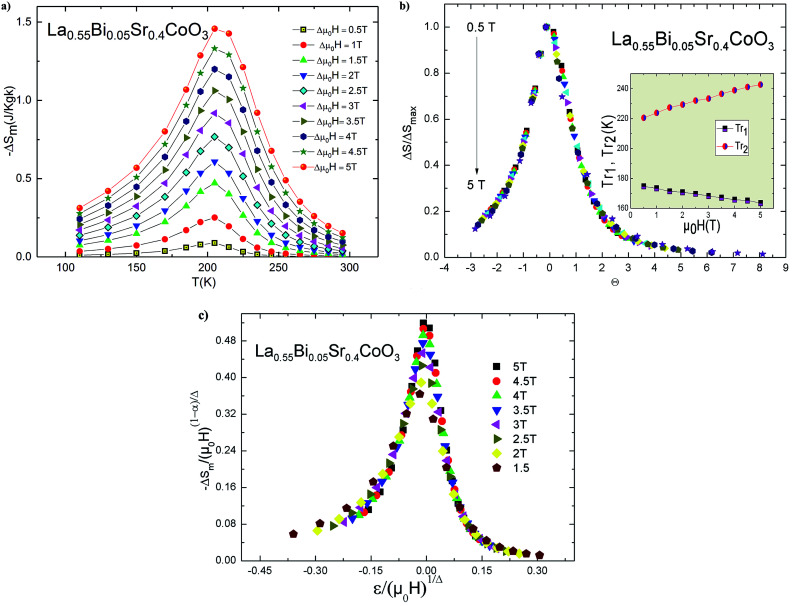
(a) Temperature and field dependence of the magnetic entropy change for La_0.55_Bi_0.05_Sr_0.4_CoO_3_. (b) Universal behavior of the scaled entropy change curves under the influence of several magnetic fields. Inset: the dependencies of the two reference temperatures (*T*_r1_ and *T*_r2_) at various magnetic fields. (c) Scaled magnetic entropy change *vs.* scaled temperature using the critical exponents.

Fig. 8a shows the behavior of Δ*S*_m_ for the La_0.55_Bi_0.05_Sr_0.4_CoO_3_ sample as a function of temperature. These curves exhibit peaks around *T*_C_. Immediately below and above *T*_C_, the −Δ*S*_m_ value monotonically increases with an increasing magnetic field, which corresponds to a magnetic FM–PM transition. The dependence of the magnetic entropy changes on the value of (∂*M*/∂*T*)_*H*_ has been clearly indicated in eqn (8). Therefore, a large magnetic entropy change usually occurs near *T*_C_, where the magnetization changes swiftly with a variation in temperature. Therefore, the negative sign of the magnetic entropy change confirms the FM character of our sample.^47^ The large values of −Δ*S*_m_ for the La_0.55_Bi_0.05_Sr_0.4_CoO_3_ system are due to a second-order magnetic transition.^48^ The magnitude of Δ*S*_m_ increases with an increasing strength of *μ*_0_*H*. The maximum value of Δ*S* decreases from 2.66 J kg^−1^ K^−1^ for the La_0.6_Sr_0.4_CoO_3_ sample prepared by the solid–solid reaction^49^ (2.10 J kg^−1^ K^−1^ for La_0.6_Sr_0.4_CoO_3_ made by the sol–gel method^14^) to 1.45 J kg^−1^ K^−1^ under an applied magnetic field of 5 T. This indicates that Bi substitution leads to a marginal decrease in the MCE properties with a reduced transition temperature. On the other hand, the obtained value of Bi-doped cobaltite is comparable to those obtained in other cobaltites,^3,14,50,51^ indicating that our sample could be used as a refrigerant material in magnetic cooling devices.

In order to determine the magnetic refrigeration efficiency, only the magnitude of the magnetic entropy is insufficient. The relative cooling power (RCP) is another decisive parameter that can be used to select materials for practical applications. RCP can be calculated by the following expression:

10 RCP = |Δ*S*^max^_m_| × δ*T*_FWHM_

where δ*T*_FWHM_ is the full width at half maximum of Δ*S*_M_(*T*). For *μ*_0_*H* = 5 T, the RCP is ∼115.5 J kg^−1^, which is of the same order of magnitude as those found in other cobaltites, such as La_0.6_Sr_0.4_CoO_3_, La_0.5_Sr_0.5_CoO_3_, and La_2/3_Sr_1/3_CoO_3_.^49,50,52^ Evidently, substituting La^3+^ by Bi^3+^, Δ*S*_M_(*T*) shows a considerably broad variation with temperature around *T*_C_ as compared with the parent sample. Such an effect is beneficial for magnetic refrigeration.

Moreover, we can use the obtained Δ*S*_m_ curves to accurately distinguish between the order of the PM–FM phase transition according to a phenomenological universal curve of the field dependence of magnetic entropy change.^53^ Such a method has been successfully applied to FM perovskites, such as cobaltites^14,54^ and manganites.^55–58^ The universal curve could be plotted by means of the normalized entropy change (Δ*S*_m_/Δ*S*^peak^_m_) and rescaling temperature (*θ*) as follows:^53^11
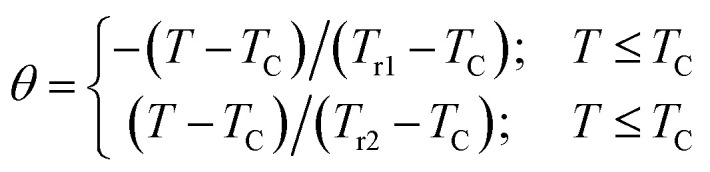
where *T*_r1_ and *T*_r2_ denote the temperatures of the two reference points that were selected as those corresponding to Δ*S*^peak^_m_/2. If all the universal curves of Δ*S*_m_(*θ*) at various magnetic fields collapse onto a single universal curve, the nature of the second-order transition would be confirmed. As shown in Fig. 8b, it is evident that all the (Δ*S*_m_/Δ*S*^peak^_m_) values fall onto one universal curve, which is consistent with the analysis based on the Arrott plots. The field dependencies of *T*_r1_ and *T*_r2_ for the investigated sample are shown in the inset of Fig. 8b.

Based on the relationship between the critical exponents and the scaled equation of state^59,60^ defined as 
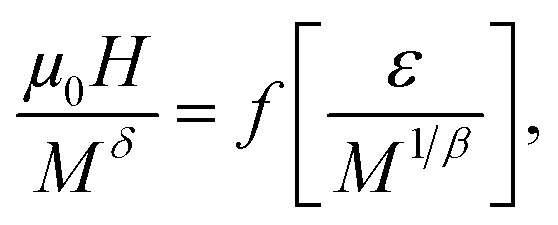
 the Δ*S*_m_ value can be described in the form of the following scaling relationship:^17^12
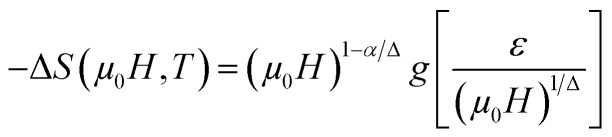
where *α* and *Δ* are the usual critical exponents, which can be obtained by using *Δ* = *β* + *γ* and *α* + 2*β* + *γ* = 2.^61^ The latter relation has been successfully applied to several manganite systems.^62,63^ This scaling relation is used to confirm the validity of the estimated critical exponents for La_0.55_Bi_0.05_Sr_0.4_CoO_3_. Fig. 8c shows that all the Δ*S*_m_(*T*) values fall on a universal curve for several applied magnetic fields. The excellent overlap of the experimental data points confirms that the estimated critical exponents and *T*_C_ for the investigated compound are in obedience with the scaling theory.

**Fig. 9:**
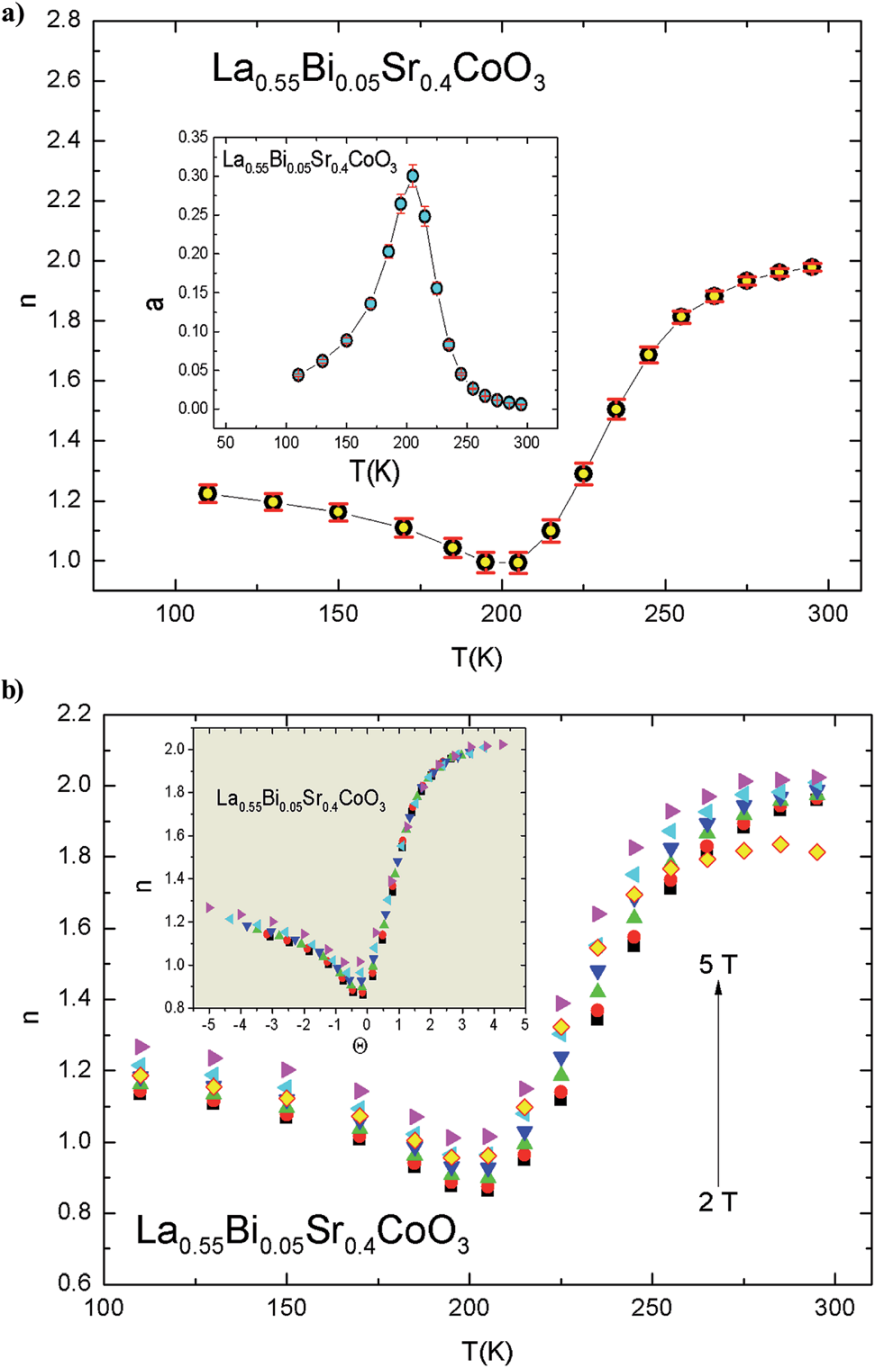
(a) Temperature dependence of exponent *n*. Inset: parameter a *vs.* temperature. (b) Local exponent *n* at several magnetic fields for the La_0.55_Bi_0.05_Sr_0.4_CoO_3_ compound. Inset: *θ* dependence of the local exponent *n*.

Based on the MCE data, the dependence of the magnetic entropy change on the external magnetic field is analyzed. Δ*S*_m_ can be expressed as a power law of the following form:

13 Δ*S*_max_(*T*, *μ*_0_*H*) = *a*(*T*)(*μ*_0_*H*)^*n*(*T*,*H*)^

where “*a*” depends on the temperature and exponent “*n*” depends, in general, on both the temperature and field. Fig. 9a shows the temperature dependencies of the exponents a (*T*) and *n*(*T*), which were determined by using eqn (13) from the Δ*S*_m_ values at several magnetic fields. From Fig. 9a, it is evident that the value of a (*T*) increases with the temperature, reaching the value of ≈0.3 at 210 K. This value is very low as compared to those observed in manganite systems.^63,64^ The value of *n* reaches the minimum of *n* = 0.989 at *T*_C_ = 210 K.

Basically, the MFT predicts that for materials with SOPT, the *n*(*T*) curve exhibits three regimes: well below *T*_C_ (*n* = 1), well above *T*_C_ (*n* = 2), and at *T*_C_ (*n* = 2/3, which is related to the critical exponents of the transition).^65^ The exponent “*n*” depends on both temperature and field and can be locally estimated using the following formula:^66^14
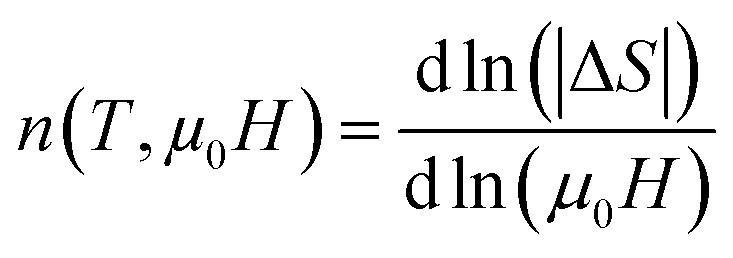


From the curves of *n*(*T*, *μ*_0_*H*) shown in Fig. 9b for the La_0.55_Bi_0.05_Sr_0.4_CoO_3_ compound, it is evident that all the curves exhibit the minimum value of *n* at *T*_C_, which is different from the MFT value of *n* = 2/3. In addition, we observed that the *n* values are unstable under varying temperature *T* and field *μ*_0_*H*. The minima of the curves with changes in *T*_C_ with the applied magnetic field range within 0.861–1.021. This behavior is related to the magnetic disorder and FM clusters in the vicinity of *T*_C_.^14,67,68^ A similar behavior has been previously reported in other perovskites.^36,63,69,70^ In order to verify the collapse or breakdown of *n*(*T*, *μ*_0_*H*) curves under the influence of different applied fields, we first arbitrarily selected the reference temperatures (*T*_r_) as those that have *n*(*T*_r_) = 1.5 (ref. 55) and constructed *n*(*θ*, *μ*_0_*H*) as a function of the rescaled temperature *θ*, which is, in turn, obtained as follows:

15 *θ* = (*T* − *T*_C_)/(*T*_r_ − *T*_C_)

From the inset of Fig. 9b, it is evident that all the *n*(*θ*, *μ*_0_*H*) values collapse onto a single universal curve, revealing a universal behavior in La_0.55_Bi_0.05_Sr_0.4_CoO_3_.

Using the MCE data, we calculated the specific heat (Δ*C*_P_) of La_0.55_Bi_0.05_Sr_0.4_CoO_3_ by means of the first derivative of Δ*S*_m_ with respect to temperature:16
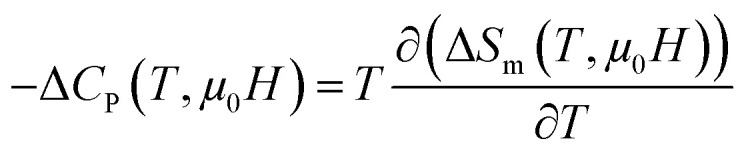


**Fig. 10:**
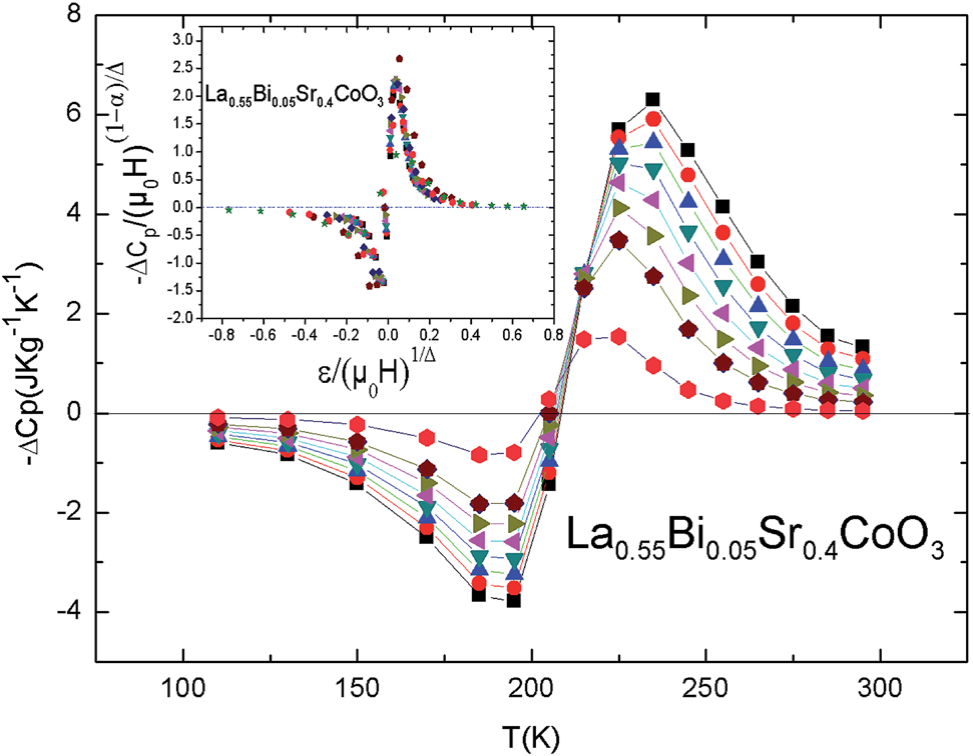
Δ*C*_p_ for the La_0.55_Bi_0.05_Sr_0.4_CoO_3_ system. Inset: normalized heat capacity change as a function of the rescaled temperature for all the magnetic fields.

Fig. 10 shows the Δ*C*_P_ value of the compound *vs.* temperature under different magnetic fields. Evidently, the Δ*C*_P_ curves represent the alternative changes from negative to positive around *T*_C_ with a negative value below *T*_C_ and a positive value above *T*_C_, which can be attributed to the FM–PM transition. This behavior has also been observed in other FM systems.^56,71–73^ The sum of the two parts is the magnetic contribution to the total of Δ*C*_P_, which has an impact on the heating or cooling power of the magnetic cooling devices.^74^ Δ*C*_P_ has the advantage of delivering values necessary to select a refrigerant material, which can simplify the design of a magnetic refrigerator.

The Δ*C*_P_ values induced by the applied magnetic fields can be plotted onto a universal curve by means of the critical exponents. The scaling method is a result of the scaling hypothesis for FM materials near their magnetic transitions. The scaling of Δ*C*_P_ changes are plotted in terms of 
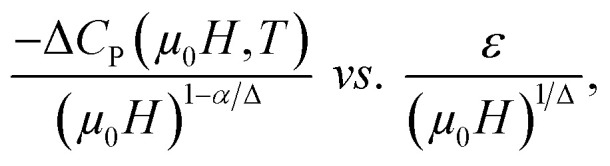
 as shown in the inset of Fig. 10. The worthwhile overlap of the data points obviously suggests that the obtained exponents (*β* and *γ*) and *T*_C_ for the La_0.55_Bi_0.05_Sr_0.4_CoO_3_ sample are in agreement with the scaling hypothesis at various magnetic fields.

Moreover, the obtained magnetic entropy change is used to deduce the spontaneous magnetization in the La_0.55_Bi_0.05_Sr_0.4_CoO_3_ sample. According to the MFT and relationship between the magnetic entropy (*S*) and magnetization (*M*), *S*(*σ*) can be expressed as follows:^75,76^17
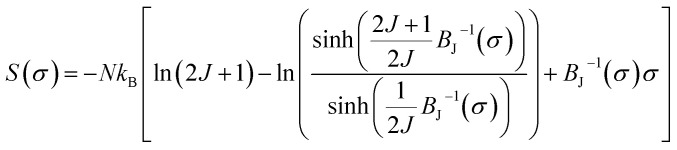
where *M* is the magnetization, *N* is the number of spins, *J* is the electron spin number, *σ* = *M*/*gμ*_B_*JN* is the reduced magnetization, *k*_B_ is the Boltzmann constant, and *B*_J_ is the Brillouin function. For small *M* values, Δ*S* is proportional to *M*^2^:^75^18
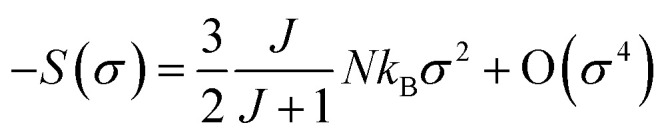


Furthermore, it should be noted that the compound exhibits spontaneous magnetization below *T*_C_ (FM state), and consequently, the *σ* = 0 state is certainly not obtained. By considering only the first term of eqn (18), Δ*S*_m_ may be expressed as19
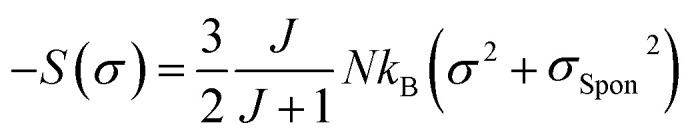


**Fig. 11:**
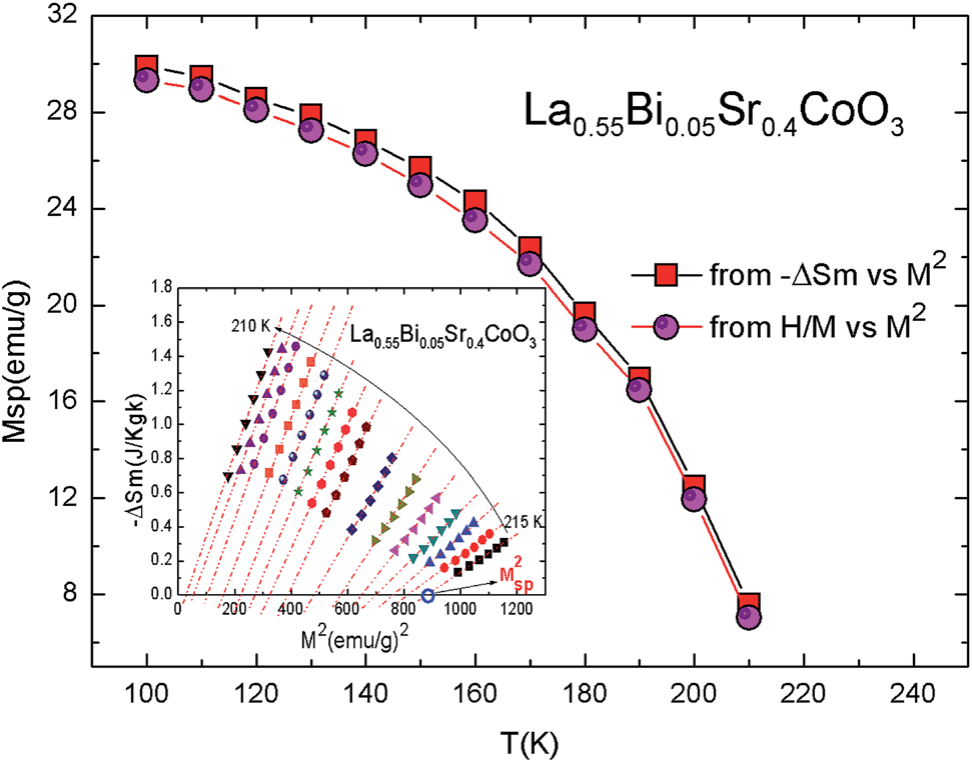
Spontaneous magnetization of La_0.55_Bi_0.05_Sr_0.4_CoO_3_ determined from the extrapolation of −Δ*S*_m_*vs. M*^2^ curves and from the mean-field results. Inset: −Δ*S*_m_*vs. M*^2^ curves. The dashed lines are linear fits to the data.

The latter equation indicates that Δ*S*_m_*vs. M*^2^ plots show a linear variation with a constant slope in the FM region. At different temperatures, all the curves exhibit a horizontal drift from the origin corresponding to a value of *M*_spon_^2^(*T*). For the PM region, the Δ*S*_m_*vs. M*^2^ plots start at a null *M* value.^77^Fig. 11 shows the *M*_sp_(*T*) data obtained from the Δ*S*_m_*vs. M*^2^ curves by the intersection of the linearly extrapolated curve with the *M*^2^ axis (inset, Fig. 11). The linear behavior of −Δ*S*_m_*vs. M*^2^ confirmed the validity of the linear expansion of eqn (19). In the same figure, we show a comparison between the estimated *M*_sp_(*T*) values obtained from the isothermal (−Δ*S*_m_) *vs. M*^2^ plots and those obtained from the Arrott plots (*μ*_0_*H vs. M*^2^). The worthwhile agreement between the aforementioned methods confirms the validity of this process in order to determine the *M*_sp_ value using a mean-field analysis of Δ*S*_m_ in the La_0.55_Bi_0.05_Sr_0.4_CoO_3_ system. The magnetic behavior of our sample is effectively described by the classical MFT.

Finally, the investigation of the scaling hypotheses of the thermomagnetic properties of the La_0.55_Bi_0.05_Sr_0.4_CoO_3_ sample offer the opportunity of using the universal curve in the investigations of novel FM materials on various applied functionalities. Such methods present a simple screening procedure of the performance of FM compounds, simple way to extrapolate results to magnetic fields or temperatures not available in the laboratories, remake the impact of non-saturating conditions, reduce experimental noise, or eliminate the effects of minority magnetic phases.

## 5. Conclusion

In summary, we conducted an in-depth investigation on magnetic, critical behavior, and MCE effect on La_0.55_Bi_0.05_Sr_0.4_CoO_3_ cobaltite synthesized *via* the solid-state reaction. The XRD analysis shows that the sample exhibits a rhombohedral structure with the *R*3̄*c* space group. The magnetic properties reveal that our sample undergoes a FM–PM second-order magnetic transition, which can be confirmed from the Arrott curves and universal curves of magnetic entropy changes. The critical behavior of the La_0.55_Bi_0.05_Sr_0.4_CoO_3_ cobaltite is studied by means of various techniques and validated by using the scaling theory and Widom scaling relation. The obtained critical exponents are close to the theoretical prediction of the mean-field model values, which implies that long-range interactions dominate the critical behavior in La_0.55_Bi_0.05_Sr_0.4_CoO_3_. Moreover, the experimental magnetic entropy changes, specific heat capacity changes, and local exponents *n* obtained for several magnetic fields collapse onto a universal curve, confirming the universal behavior of the MCE properties in this oxide. The estimated spontaneous magnetization value extracted through the analysis of the magnetic entropy change (−Δ*S*_m_*vs. M*^2^) is consistent with that extracted through the classical extrapolation of the Arrott plots (*μ*_0_*H*/*M vs. M*^2^). Consequently, the magnetic entropy change is a valid approach to determine the spontaneous magnetization of the La_0.55_Bi_0.05_Sr_0.4_CoO_3_ compound.

## Conflicts of interest

There are no conflicts to declare.

## Supplementary Material
